# Global pattern of cardiovascular disease management in patients with cancer and impact of COVID-19 on drug selection: IRAQ—IC-OS survey-based study

**DOI:** 10.3389/fcvm.2022.979631

**Published:** 2022-09-21

**Authors:** Hasan Ali Farhan, Israa Fadhil Yaseen, Mohammed Alomar, Daniel Lenihan, Susan Dent, Alexander R. Lyon

**Affiliations:** ^1^Scientific Council of Cardiology, Iraqi Board for Medical Specializations, Baghdad, Iraq; ^2^Baghdad Heart Center, Baghdad Teaching Hospital, Medical City, Baghdad, Iraq; ^3^University of South Florida, H. Lee Moffitt Cancer Center and Research Institute, Tampa, FL, United States; ^4^International Cardio-Oncology Society, Tampa, FL, United States; ^5^Department of Medicine, Duke Cancer Institute, Duke University, Durham, NC, United States; ^6^National Heart and Lung Institute, Imperial College, London, United Kingdom; ^7^Cardio-Oncology Service, Royal Brompton Hospital, London, United Kingdom

**Keywords:** cardio-oncology, cardiovascular disease, COVID-19, hypertension, heart failure, anticoagulant

## Abstract

**Background:**

Regional variations in cardiovascular disease (CVD) and CVD management are well known. However, there is limited information on geographical variations in the discipline of Cardio-Oncology, including both the nature of CVD in patients with cancer and its management. Furthermore, during the recent COVID-19 pandemic, CV care for patients was disrupted resulting in an unknown impact on cardio-oncology services.

**Objective:**

The aim of this study was to identify the regional variations in the management of CVD among patients with cancer and the impact of the COVID-19 pandemic on the selection of cardiovascular drugs in cardio-oncology.

**Methods:**

An online survey was conducted by the Iraq Chapter of the International Cardio-Oncology Society (IC-OS). The survey was shared with cardiologists and oncologists in all seven continents to identify whether regional variations exist in cardio-oncology daily practice.

**Results:**

From April to July 2021, 140 participants responded to the survey, including cardiologists (72.9%) and oncologists (27.1%). Most of the respondents were from the Middle East (26.4%), North America (25%), Latin America and the Caribbean (25%), and Europe (20.7%). Baseline CV risk assessment in patients with cancer using the HFA/IC-OS score was reported in 75.7% of respondents (78.4% cardiologists and 68.4% oncologists). Hypertension was the most common CVD treated by the survey respondents globally (52.1%) unlike in Europe where heart failure was the most prominent CVD (51.7%). The blood pressure cutoff value to initiate hypertension management is >140/90 mmHg globally (72.9%), but in North America (48.6%) it was >130/80 mmHg. In the Middle East, 43.2% of respondents do not use cardioprotective medication. During the COVID-19 pandemic, 10.7% of respondents changed their practice, such as switching from prescribing ACEI to ARB. Apixaban is the main anticoagulant used in patients with cancer (32.9%); however, in cancer patients with COVID-19 infection, the majority used enoxaparin (31.4%).

**Conclusion:**

More than three-quarters of cardiologists and oncologists responding to the survey are using HFA/IC-OS proformas. The survey showed regional variations in the management of CVD on different continents. The use of cardioprotective agents was limited in some regions including the Middle East. COVID-19 pandemic impacted daily practice on the selection and switching of cardiovascular drugs including ACEI/ARB and the choice of anticoagulants.

## Introduction

Regional variations in the prevalence of cardiovascular (CV) morbidity and mortality in different continents had been well documented (1). These variations can be explained by the local differences in CV risk factors and healthcare access, in addition to disparities in the implementation of guideline-directed medical therapy in clinical practice, and disparities in conduction and publication of clinical research with the highest rates in the high-income countries (81.1%) (1–3). Among patients with cancer, the time of diagnosis and treatment of CV disease (CVD) have different patterns. One study reported that 11% of patients with cancer had baseline CVD and a further 16% were diagnosed with new CVD at the time of their cancer diagnosis up to 5 years of post-cancer diagnosis (4). To reduce CV complications associated with cancer therapies, the Heart Failure Association (HFA) of the European Society of Cardiology and the International Cardio-Oncology Society (IC-OS) designed seven baseline CV risk stratification proformas for the most common cardiotoxic cancer therapies. Risk scores were developed for each class of cancer therapy with recommendations on CVD management strategies based on individual risk (5). This study was designed to report the international variations in the pattern of CVD and CVD management in patients with cancer. A secondary objective was to evaluate the impact of the COVID-19 pandemic on the selection of CV drugs among patients with cancer.

## Methods

### Study design

An online survey was conducted in collaboration with the Iraq Chapter of IC-OS and the international leadership of IC-OS (see Supplementary Table 1). The survey included 18 multiple-choice questions. Questions included the respondents' demographics, patient baseline risk stratification, cardiotoxicity monitoring, management of CVD in patients with cancer, and choice of CV drugs to treat CVD before and during the COVID-19 pandemic. Continents were included in the demographic questions to assess regional variations in cardio-oncology. Google Form (Google Corporation, Mountain View, California, USA) was used. IC-OS shared the survey's link by emails with its members, who are experts or have an interest in cardio-oncology, with two reminder emails. In addition, social media including WhatsApp (WhatsApp Inc., Menlo Park, California, USA) and Viber (Rakuten Inc., Tokyo, Japan) applications were used to share the link with potential respondents. For the survey validation, the questions were assessed initially by the authors for simplicity and clarification. Then, a pilot test was performed by sharing the link of the survey with the 16 presidents of IC-OS chapters, and the required amendments were completed. Responding to the survey by the participants was voluntary. To avoid responding to the survey more than one time by the same participant, there was a mandatory question at the end of the survey to mention the responders' emails (Supplementary Table 1). Before data analysis of the results, it was double-checked for multiple responses from the same participant. When multiple responses were noticed from the same participant, only the first response was included in the data analysis.

### Statistical analysis

Categorical variables were presented as numbers and percentages. Excel for Mac, version 15.13.3 was used for statistical analysis.

## Results

**Figure 1:**
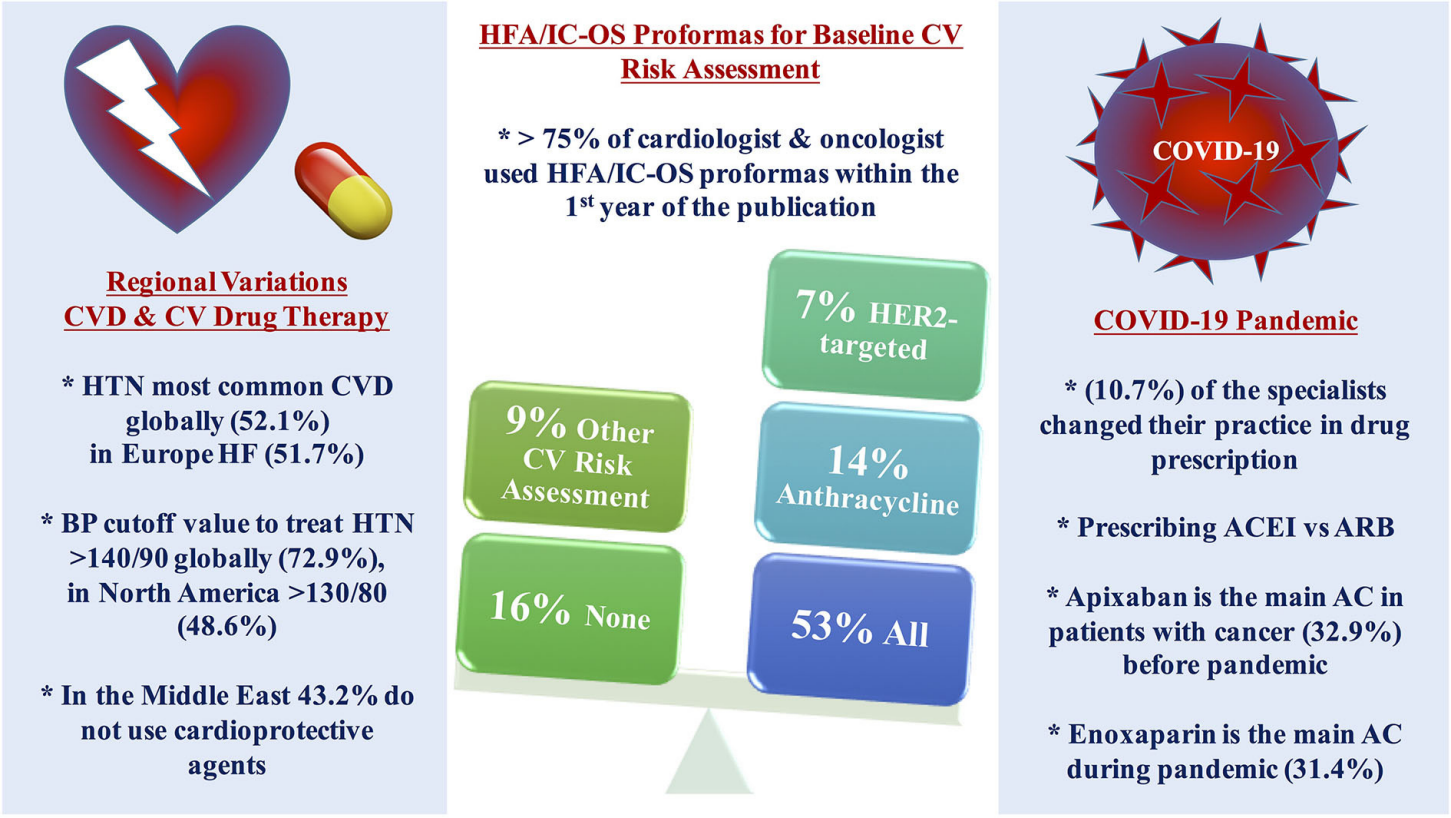
Central illustration of the main results. More than three-quarters of the cardiologists and oncologists are using HFA/IC-OS proformas for baseline CV risk stratification among patients with cancer, more than half of the respondents use all the proformas, while using only one proforma was found mainly with anthracycline followed by HER2-targeted therapy with the limited use of other proformas. Regional variations were found in the pattern of the main baseline CVD and CV management among patients with cancer in different continents. The use of cardioprotective agents is limited in the Middle East. COVID-19 pandemic impacted on the decision of selection between CV drug therapy and anticoagulant. AC, anticoagulant; ACEI, angiotensin-converting enzyme inhibitor; ARB, angiotensin receptor blocker; CV, cardiovascular; CVD, cardiovascular disease; HF, heart failure; HFA/IC-OS, Heart Failure Association/International Society of Cardio-Oncology; HTN, hypertension; LMWH, low molecular weight heparin.

Responses to the survey occurred between April 2021 and July 2021, 140 participants responded globally, and 78.6% of them are IC-OS members. Main results of the survey are shown in Figure 1.

### Demographics of the respondents

The majority of respondents (67.9%) were cardiologists, 26.4% from the Middle East, 25% from North America, 25% from Latin America and the Caribbean, and 20.7% from Europe (Table 1).

**Table 1:** Demographics of the survey responders.

**Characteristics**	**No. (%)**		
**Specialty**	**Overall 140 (100)**	**Cardiologist 102 (72.9)**	**Oncologist 38 (27.1)**
Cardiology	Cardiologist	95 (67.9)		
	Cardiology resident/fellow	7 (5)		
Oncology including haemato-oncology	Oncologist	32 (22.9)		
	Oncology resident/fellow	3 (2.1)		
	Radiation Oncologist	3 (2.1)		
**Region of current practice**	
Africa	1 (0.7)	0 (0)	1 (2.6)
Asia	2 (1.4)	1 (1)	1 (2.6)
Europe	29 (20.7)	28 (27.5)	1 (2.6)
Latin America and the Caribbean	35 (25)	33 (32.4)	2 (5.3)
Middle East	37 (26.4)	8 (7.8)	29 (76.3)
North America	35 (25)	31 (30.4)	4 (10.5)
Oceania	1 (0.7)	1 (1)	0 (0)
Years of practice
1–5 years	35 (25)	29 (28.4)	6 (15.8)
6–10 years	31 (22.1)	19 (18.6)	12 (31.6)
>10 years	74 (52.9)	54 (52.9)	20 (52.6)
**Prescribing of cardiovascular drugs for patients with cancer**	
Yes	128 (91.4)	100 (98)	28 (73.7)
No	12 (8.6)	2 (2)	10 (26.3)

### Baseline risk stratification and drug monitoring

**Figure 2:**
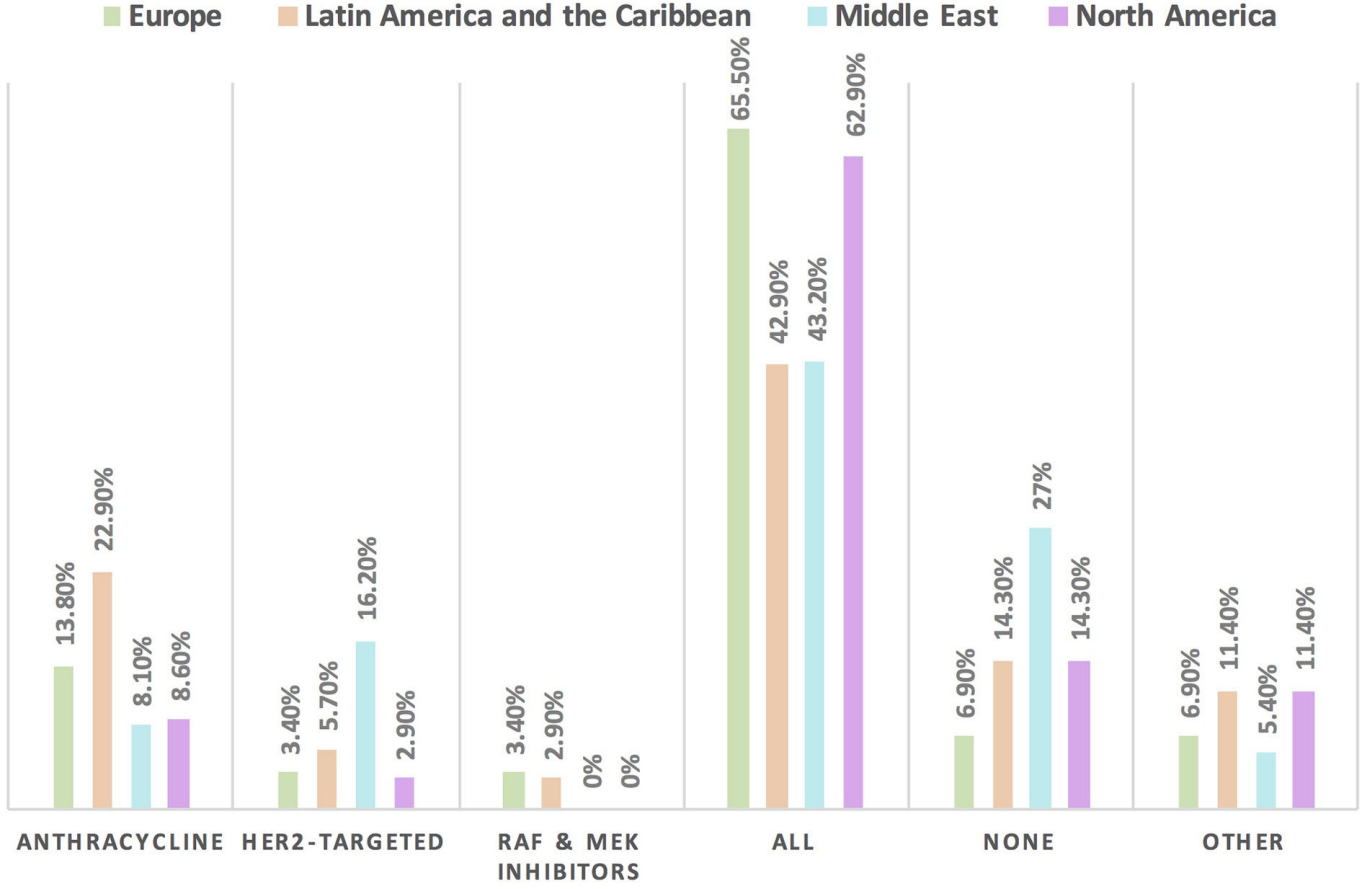
Frequency of using HFA/IC-OS proformas for baseline CVD risk stratification in cardio-oncology in different continents. The highest rate for the use of all HFA/IC-OS proformas for baseline cardiovascular risk stratification was found in Europe followed by North America. However, the largest proportion of physicians who do not use any of these proformas was found in the Middle East.

The HFA/IC-OS proformas were used by 75.7% of respondents [cardiologists (78.4%) and oncologists (68.4%)]. All the seven HFA/IC-OS proformas were used by 52.9% of respondents mainly by cardiologists (57.8%) vs. oncologists (39.5%). Approximately 16% of respondents do not use any of these proformas, with 8.6% of responders either performing baseline risk assessments without using HFA/IC-OS proformas or sending patients for cardiology consultation. There were regional variations in the use of HFA/IC-OS proforma with the highest rate for using all the proformas in Europe (65.5%) followed by North America (62.9%), while the lowest rate is in the Middle East (27%) (Figure 2). Regarding CV drugs prescribed for patients with cancer to treat CVD or cardiotoxicity, it was found that 6% of respondents do not monitor these drugs during follow-up by sending patients for ECG, renal function test, and/or serum electrolytes. Approximately 89% of respondents check drug–drug interactions between CV drugs and cancer therapies before prescribing CV drugs (Table 2).

**Table 2:** Baseline cardiovascular risk stratification, drug monitoring, and drug interaction in cardio-oncology.

**Risk Stratification/monitor**	**No. (%)**		
**HFA/IC-OS proformas**	**Overall 140 (100)**	**Cardiologist 102 (72.9)**	**Oncologist 38 (27.1)**
Anthracycline chemotherapy	19 (13.60)	15 (14.7)	4 (10.5)
HER2-targeted cancer therapies	10 (7.10)	4 (3.9)	6 (15.8)
VEGF inhibitors	0 (0)	0 (0)	0 (0)
Multi-targeted kinase inhibitors for CML	1 (0.70)	0 (0)	1 (2.6)
PIs and immunomodulatory agents for multiple myeloma	0 (0)	0 (0)	0 (0)
Combination RAF and MEK inhibitors	2 (1.40)	2 (20)	0 (0)
Androgen deprivation therapies	0 (0)	0 (0)	0 (0)
All of the proformas	74 (52.90)	59 (57.8)	15 (39.5)
None	22 (15.70)	12 (11.8)	10 (26.3)
Other	12 (8.6)	10 (9.8)	2 (5.3)
**Monitoring of cardiovascular drugs**	
Yes	132 (94.3)	100 (98)	32 (84.2)
No	8 (5.7)	2 (2)	6 (15.8)
**Check drug-drug interaction**	
Yes	124 (88.60)	93 (91.2)	31 (81.6)
No	16 (11.40)	9 (8.8)	7 (18.4)

CML, chronic myeloid leukemia; HFA/IC-OS, Heart Failure Association/International Cardio-Oncology Society; PIs, proteasome inhibitors; VEGF, vascular endothelial growth factor.

### Management of CVD in cardio-oncology patients

**Figure 3:**
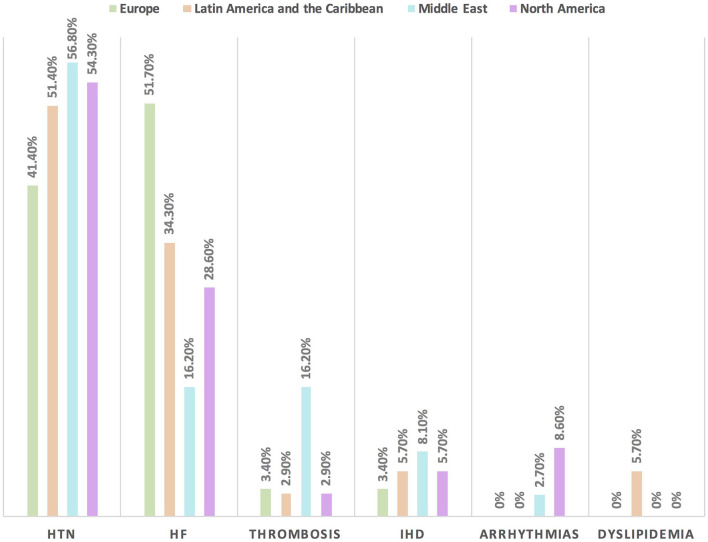
The most common CVD among patients with cancer in different continents. Hypertension is the main baseline CVD among patients with cancer in all regions except in Europe where heart failure is the most common one. HF, heart failure; HTN, hypertension; IHD, ischemic heart disease.

**Figure 4:**
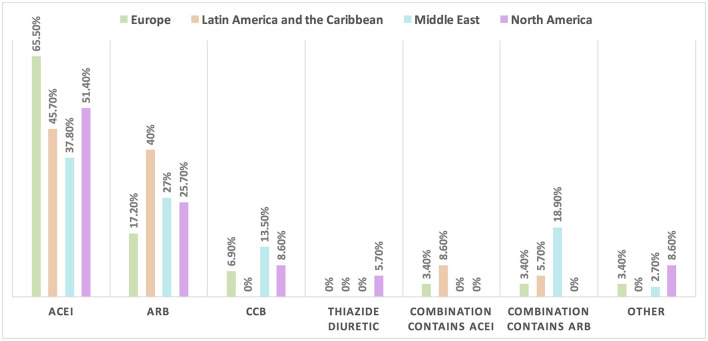
The most common treatment for hypertension not induced by cancer therapies among patients with cancer in different continents. ACEI is the main antihypertensive agent used for the management of hypertension not induced by cancer therapy. The use of combination treatment containing ARB was found most commonly in the Middle East. ACEI, angiotensin-converting enzyme inhibitor; ARB, angiotensin receptor blocker; CCB, calcium channel blocker.

**Figure 5:**
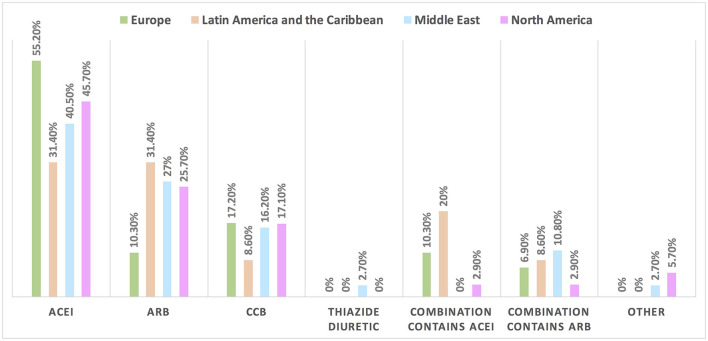
The most common treatment for hypertension induced by cancer therapies among patients with cancer in different continents. ACEI is the most commonly used drug for the management of hypertension induced by cancer therapy; however, compared with the rate of drugs used in the management of hypertension not induced by cancer therapy (in Figure 4), the rate of utilization of CCB and combination therapy increased particularly for the use of combination treatment containing ACEI in Latin America and the Caribbean. ACEI, angiotensin-converting enzyme inhibitor; ARB, angiotensin receptor blocker; CCB, calcium channel blocker.

**Figure 6:**
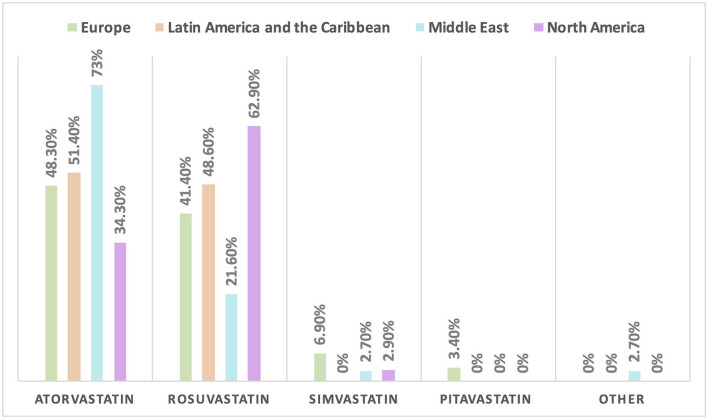
The most common statin prescribed for patients with cancer in different continents. Atorvastatin is the most commonly prescribed statin among patients with cancer, except in North America where rosuvastatin is the main statin.

**Figure 7:**
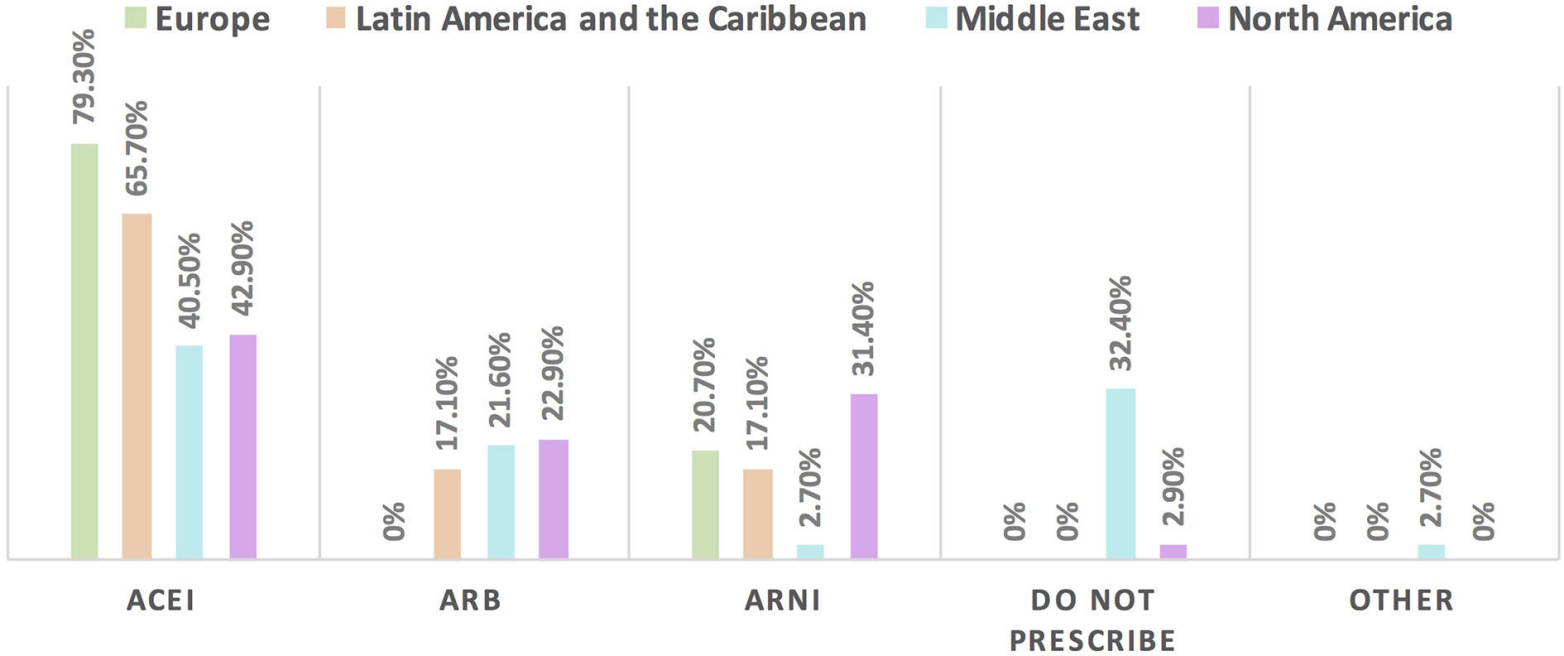
The most commonly prescribed RAAS inhibitor for patients with cancer and HFrEF in different continents. For patients with cancer and HFrEF, the main RAAS inhibitor to be used is ACEI followed by ARNI except in the Middle East where ARB is the second choice. Of note, respondents in Europe did not choose ARB among the options. One-third of the respondents from the Middle East do not prescribe RAAS may be explained by the majority of respondents in this region being oncologists. ACEI, angiotensin-converting enzyme inhibitor; ARB, angiotensin receptor blocker; ARNI, angiotensin receptor-neprilysin inhibitors; HFrEF, heart failure with reduced ejection fraction; RAAS, renin-angiotensin-aldosterone system.

**Figure 8:**
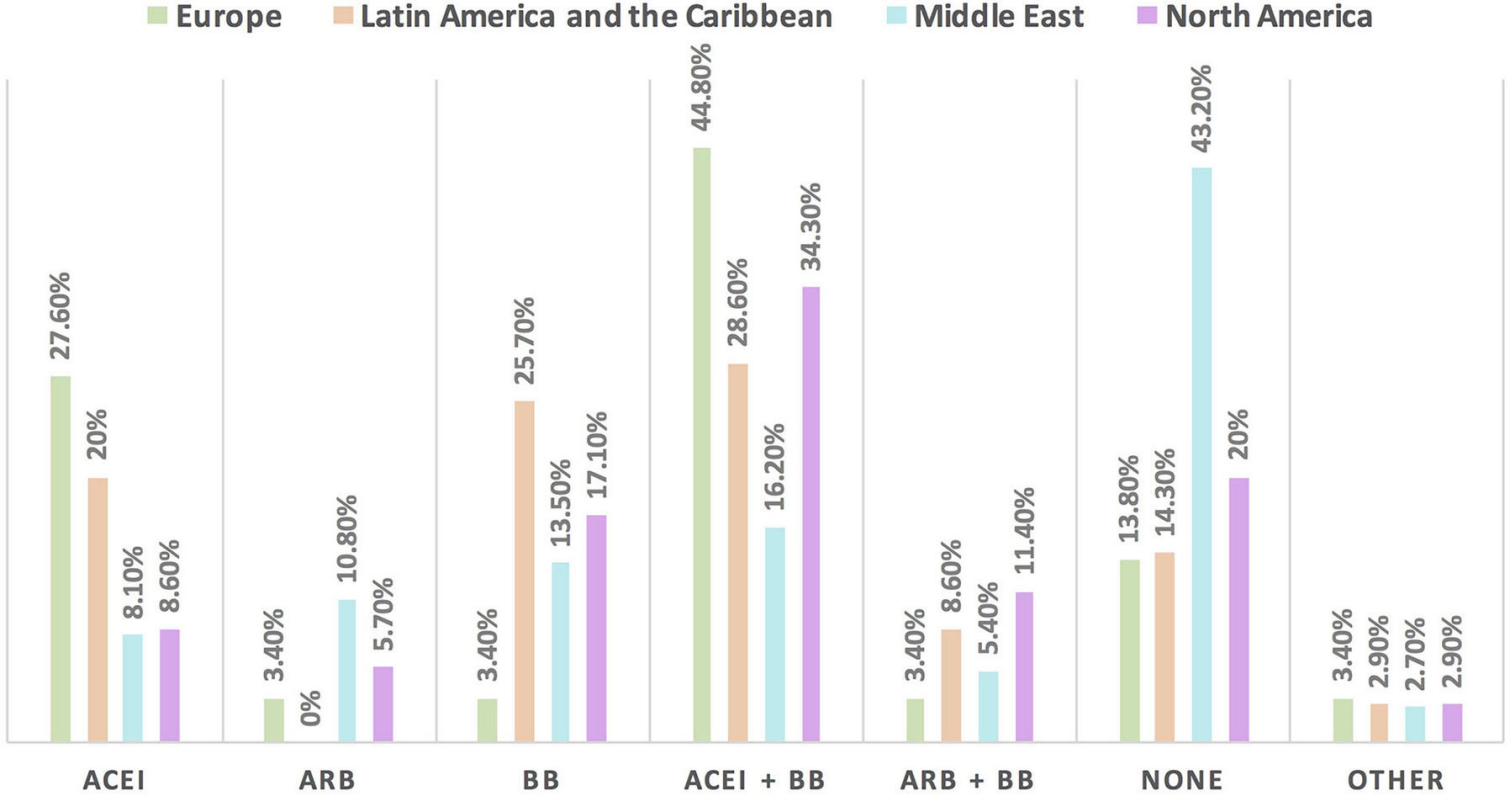
The most commonly prescribed cardioprotective agents in patients with cancer and borderline LVEF [LVEF 50%] in different continents. A combination of ACEI and BB is the most commonly prescribed cardioprotective agent in patients with cancer globally. Of note, the highest rate of respondents who do not prescribe cardioprotective agents was found in the Middle East, because most of the respondents were oncologists. ACEI, angiotensin-converting enzyme inhibitor; ARB, angiotensin receptor blocker; BB, beta blocker.

Hypertension is the most common CVD treated by the respondents (52.1%) in patients with cancer, followed by heart failure, thrombosis, ischemic heart disease, arrhythmias, and dyslipidemia. The second most common CVD to be treated by cardiologists is heart failure (41.2%), while for oncologists, it is venous thromboembolism (VTE) (18.4%) (Supplementary Table 2). In Europe, the most common CVD was heart failure (51.7%), whereas, in all other continents, hypertension was the most common one (Figure 3). The cutoff value to treat hypertension not induced by cancer therapies was >140/90 mmHg for most of the cardiologists (67.6%) and oncologists (86.8%) (Supplementary Table 2). In North America, the main cutoff value for treating hypertension is >130/80 mmHg (48.6%), whereas in all other continents, the threshold value to start treatment was >140/90 mmHg (Supplementary Figure 1). For the management of hypertension not induced by cancer therapies, angiotensin-converting enzyme inhibitors (ACEI) were the most common antihypertensive drug prescribed by cardiologists and oncologists (52.9 and 39.5%, respectively) followed by angiotensin receptor blockers (ARB) (28.4 and 26.3%, respectively). Combination prescribing, where a pill contains two or more antihypertensive drugs, for the management was used by 10.7% of the respondents, mainly by oncologists (18.4%), who tend to use a combination containing ARB rather than a pill containing ACEI (Supplementary Table 2). Regional variations have existed in prescribing antihypertensive agents. For example, calcium channel blocker (CCB) prescribing was rare in Latin America and the Caribbean respondents, and combination antihypertensives containing ACEI were prescribed neither in the Middle East nor in North America (Figure 4). On the contrary, for hypertension induced by cancer therapies, a higher percentage of cardiologists use CCB and combination antihypertensives containing ACEI (14.7 and 10.8%, respectively) for cancer therapy-induced hypertension (Supplementary Table 2). In European respondents, the second-line antihypertensive drug class after ACEI is CCB. The use of a combination of antihypertensives containing ACEI is increasing, reaching 20% in Latin America and the Caribbean but is not used again in the Middle East (Figure 5). Atorvastatin is the most commonly prescribed statin by 51.4% of survey responders, particularly the oncologists (68.4%), whereas rosuvastatin was the most frequently prescribed statin by cardiologists (52%) (Supplementary Table 2). Atorvastatin is the main statin prescribed in the Middle East (73%), Latin America and the Caribbean (51.4%), and Europe (48.3%), whereas rosuvastatin is the most commonly prescribed in North America (62.9%) (Figure 6). For patients with cancer and heart failure with reduced ejection fraction (HFrEF), ACEI is the most common renin-angiotensin-aldosterone system (RAAS) inhibitor prescribed by the respondents, followed by angiotensin receptor-neprilysin inhibitor (ARNI) (17.1%) and ARB (16.4%) (Supplementary Table 2). ARB was not prescribed in Europe for the management of HFrEF as an initial drug. In North America and Europe, ARNI is the second most common RAAS inhibitor in HFrEF management (31.4 and 20.7%, respectively) (Figure 7). The combination of ACEI and beta-blocker (BB) is the most prescribed cardioprotective strategy (30.7%) in patients with cancer and borderline left ventricular ejection fraction (LVEF 50%), prescribed by both cardiologists (35.3%) and oncologists (18.4%); however, 22.9% of respondents do not prescribe any cardioprotective medication (Supplementary Table 2). Compared with the responses from other continents, respondents of 2-fold from the Middle East do not prescribe cardioprotective medication (Figure 8).

### Impact of COVID-19 pandemic on drug selection

**Figure 9:**
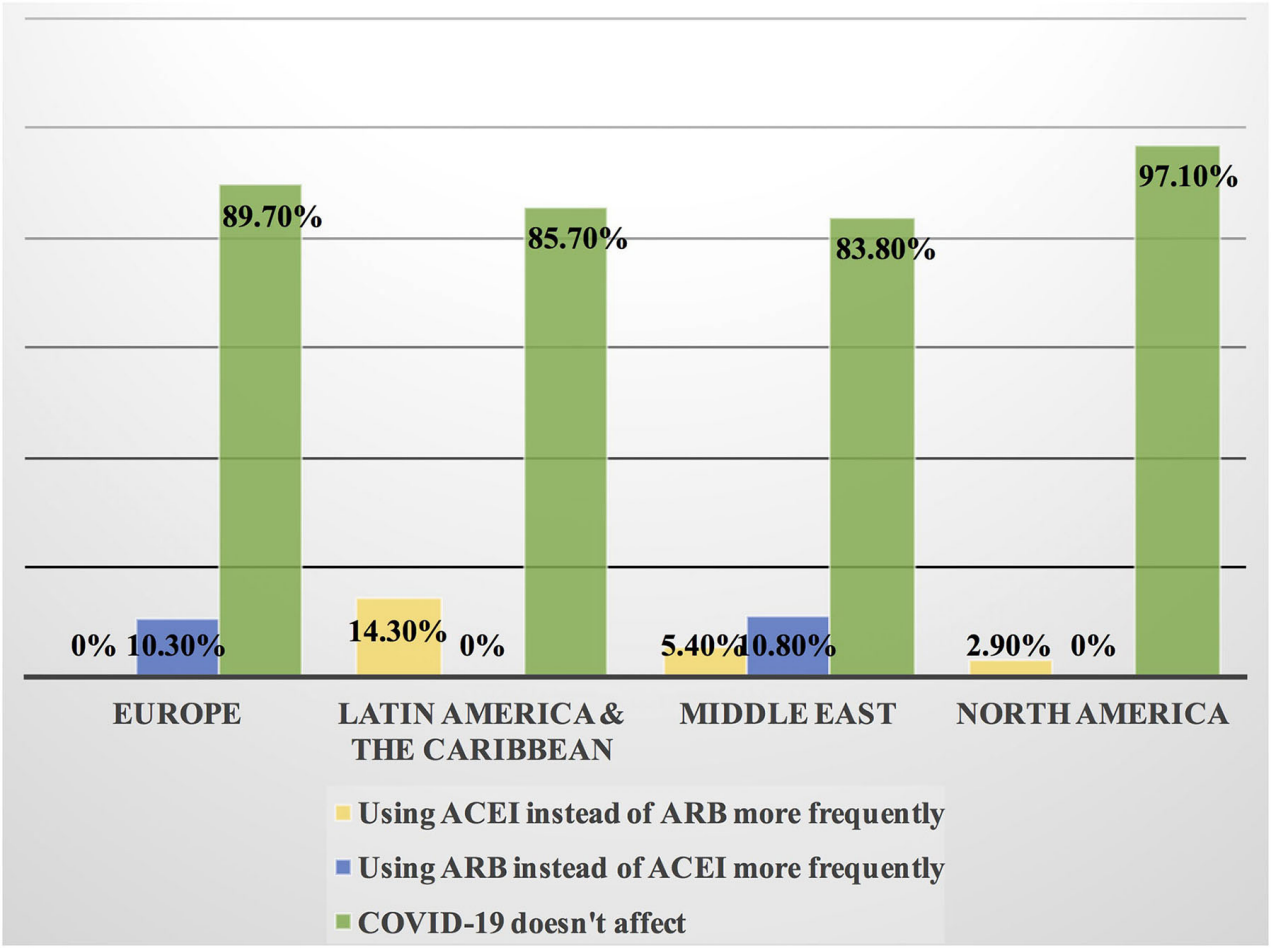
Impact of the COVID-19 pandemic on the prescribing of ACEI vs. ARB among patients with cancer in different continents. Prescribing ACEI and ARB for patients with cancer by most of the cardiologists and oncologists was not affected by the COVID-19 pandemic globally; however, the highest rate of changing practice in the management was found in Latin America and the Caribbean where ACEI was prescribed more frequently and in Europe where ARB was prescribed more than ACEI. ACEI, angiotensin-converting enzyme inhibitor; ARB, angiotensin receptor blocker.

**Figure 10:**
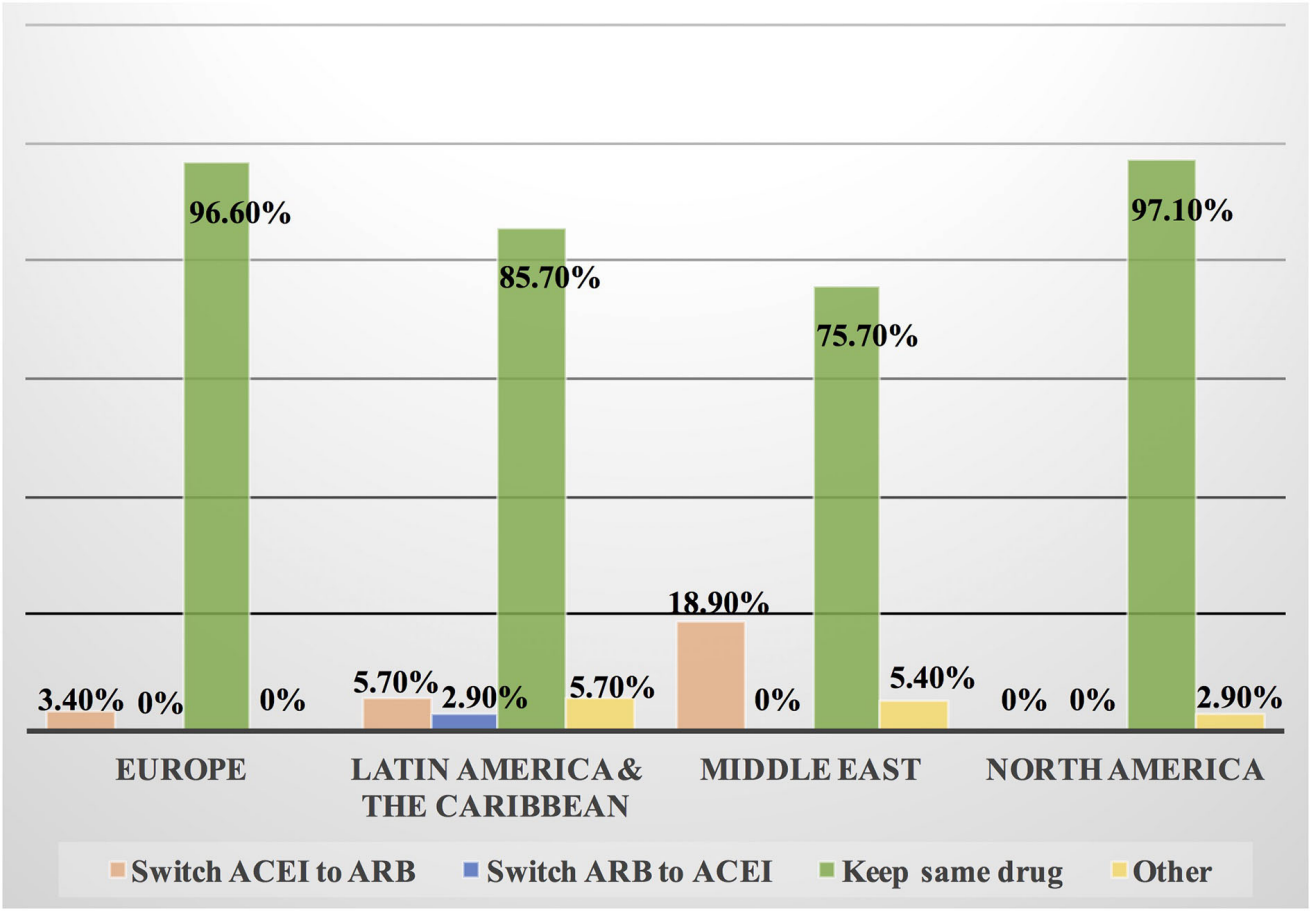
Switching between ACEI and ARB among patients with cancer and COVID-19 infection in different continents. Some cardiologists and oncologists switched ACEI to ARB during the COVID-19 pandemic, mainly in the Middle East, Latin America and the Caribbean. However, most of the respondents kept patients on the same drug whether ACEI or ARB. ACEI, angiotensin-converting enzyme inhibitor; ARB, angiotensin receptor blocker.

**Figure 11:**
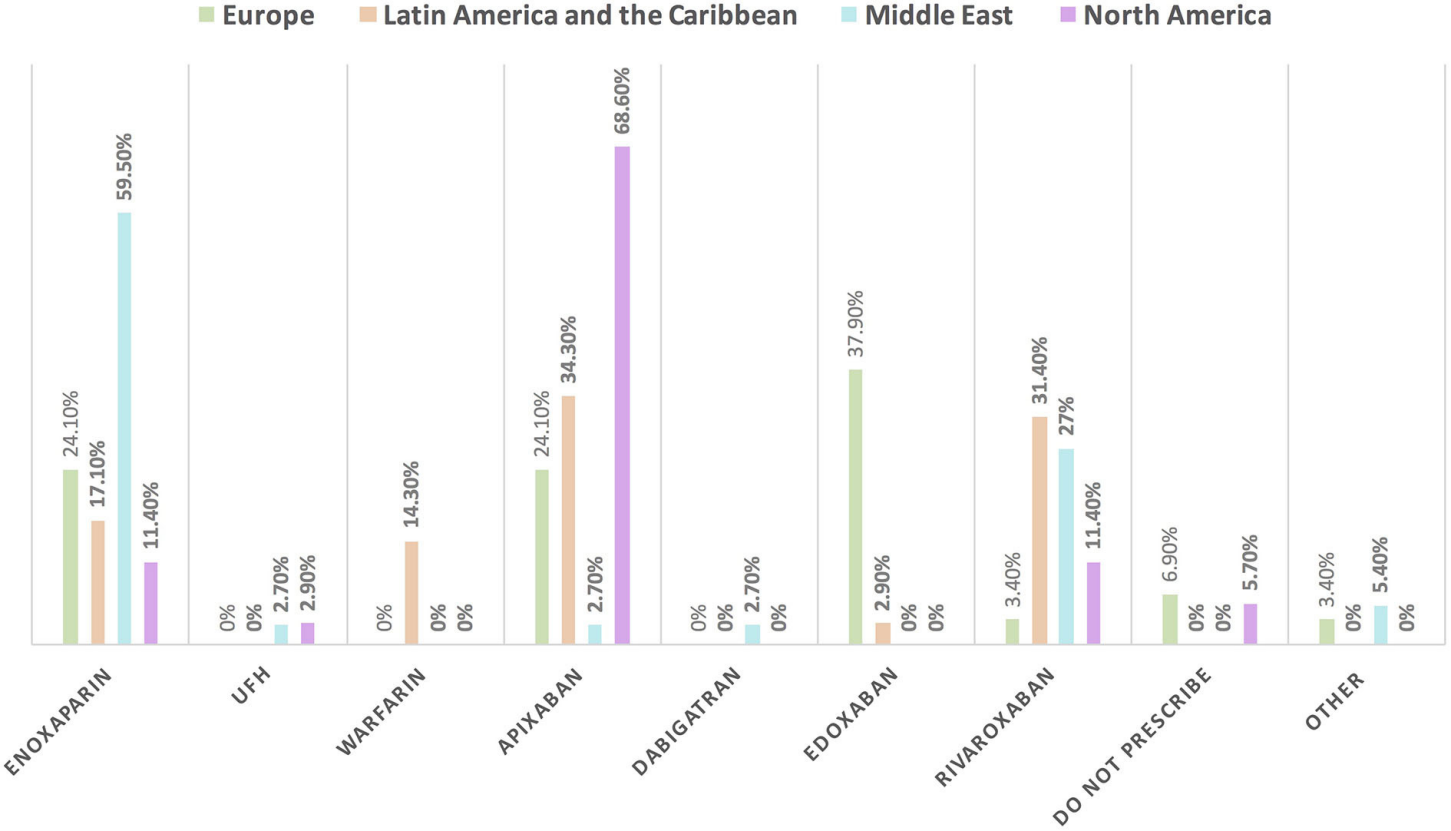
The most common prescribed anticoagulant for the management of VTE in patients with cancer without COVID-19 infection in different continents. There was obvious regional variation in the selection of anticoagulants for the management of VTE in patients with cancer. The most commonly prescribed anticoagulant is enoxaparin in the Middle East; apixaban in North America, Latin America and the Caribbean; and edoxaban in Europe. UFH, unfractionated heparin; VTE, venous thromboembolic events.

**Figure 12:**
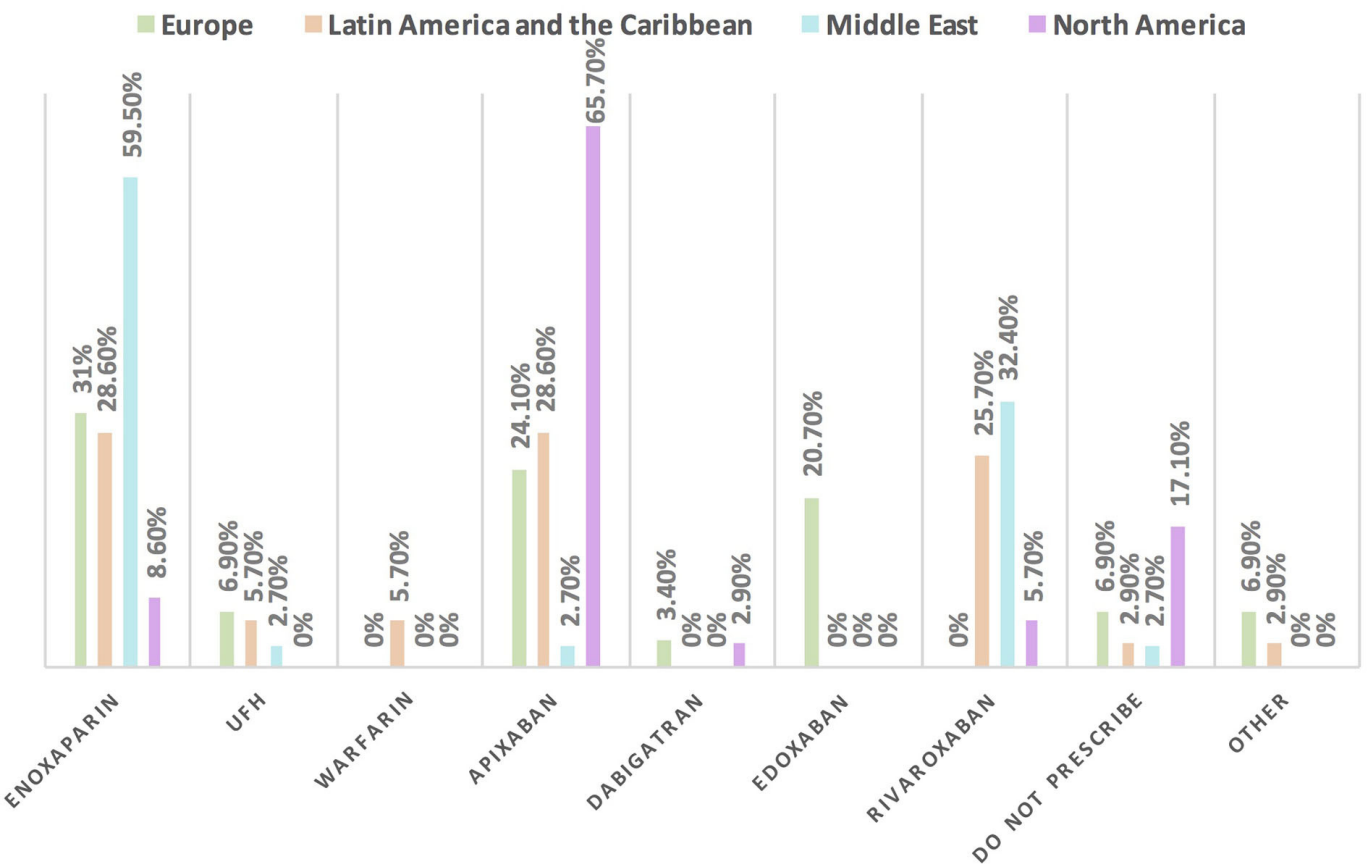
The most commonly prescribed anticoagulant for patients with cancer and COVID-19 infection in different continents. Enoxaparin is the most commonly prescribed anticoagulant in patients with cancer and COVID-19 infection, unlike in North America where apixaban is the main one. UFH, unfractionated heparin.

Approximately 11% of the respondents' decision for prescribing ACEI vs. ARB was affected by the COVID-19 pandemic, and approximately 10% switched between two drugs mainly ACEI and ARB (7.1%), particularly by oncologists (Table 3). Experts in North America, Latin America and the Caribbean did not prefer the use of ARB over ACEI during the COVID-19 pandemic, unlike the Middle East and Europe where ARB was used more than ACEI by 10.8 and 10.3%, respectively (Figures 9, 10). Regarding the use of anticoagulants among patients with cancer, apixaban is the most common anticoagulant used among patients with cancer and VTE in patients without COVID-19 infection (32.9%) [mainly by the cardiologists (43.1%)] followed by enoxaparin (27.9%) [mainly by the oncologists (52.6%)]. While in the case of COVID-positive patients, enoxaparin was the main anticoagulant to be used (31.4%) [mainly by the oncologists (55.3%)] followed by apixaban (30%) [mainly by the cardiologists (40.2%)] (Table 3). Regional variations in the selection of anticoagulants for patients with cancer without COVID-19 infection existed. Apixaban was the most selected anticoagulant in North America (68.6%), Latin America and the Caribbean. Conversely, in the Middle East, enoxaparin (59.5%) was the most commonly prescribed anticoagulant in patients with cancer according to the respondents, while edoxaban was the most commonly prescribed anticoagulant by the responders in Europe (37.9%). Warfarin was only selected in Latin America and the Caribbean (14.3%) (Figure 11). Enoxaparin was the most commonly prescribed anticoagulant (31%) for patients with cancer and COVID-19 infection, with similar regional variations noticed (Figures 11, 12).

**Table 3:** Impact of COVID-19 pandemic on the selection of cardiovascular drugs in cardio-oncology.

	**Overall 140 (100)**	**Cardiologist 102 (72.9)**	**Oncologist 38 (27.1)**
**Impact of COVID-19 pandemic on prescribing ACEI vs. ARB to treat CVD among patients with cancer**			
Using ACEI instead of ARB	8 (5.70)	5 (4.9)	3 (7.9)
Using ARB instead of ACEI	7 (5)	3 (2.9)	4 (10.5)
COVID-19 doesn't affect on prescribing of ACEI/ARB	125 (89.30)	94 (92.2)	31 (81.6)
**Switching between ACEI and ARB for patients with cancer who are already on ACEI or ARB and infected with COVID-19**			
Switch ACEI to ARB	10 (7.10)	3 (2.9)	7 (18.4)
Switch ARB to ACEI	1 (0.70)	1 (1)	0 (0)
Keep the patient on his medication whether ACEI or ARB	123 (87.90)	95 (93.1)	28 (73.7)
Other	6 (4.30)	3 (2.9)	3 (7.9)
**The most common anticoagulant prescribed for patients with cancer without COVID-19 infection**			
Enoxaparin	39 (27.90)	19 (18.6)	20 (52.6)
UFH	3 (2.10)	1 (1)	2 (5.3)
Warfarin	5 (3.60)	4 (3.9)	1 (2.6)
Apixaban	46 (32.90)	44 (43.1)	2 (5.3)
Dabigatran	1 (0.70)	0 (0)	1 (2.6)
Edoxaban	12 (8.60)	12 (11.8)	0 (0)
Rivaroxaban	26 (18.60)	17 (16.7)	9 (23.7)
Do not prescribe	6 (4.30)	4 (3.9)	0 (0)
Other	2 (1.40)	1 (1)	3 (7.9)
**The most common prescribed anticoagulant for patients with cancer and COVID-19 infection**			
Enoxaparin	44 (31.40)	23 (22.5)	21 (55.3)
UFH	6 (4.30)	4 (3.9)	2 (5.3)
Warfarin	2 (1.40)	1 (1)	1 (2.6)
Apixaban	42 (30)	41 (40.2)	1 (2.6)
Dabigatran	3 (2.10)	1 (1)	2 (5.3)
Edoxaban	6 (4.30)	6 (5.9)	0 (0)
Rivaroxaban	23 (16.40)	14 (13.7)	9 (23.7)
Do not prescribe	10 (7.10)	8 (7.8)	2 (5.3)
Other	4 (2.90)	4 (3.9)	0 (0)

ACEI, angiotensin-converting enzyme inhibitor; ARB, angiotensin receptor blocker; CVD, cardiovascular disease; UFH, unfractionated heparin.

## Discussion and conclusion

This international survey shows the pattern of practice in cardio-oncology by cardiologists and oncologists interested in the growing field of cardio-oncology. More than 75% of the respondents use at least one HFA/IC-OS baseline CV risk stratification proforma. More than half of the cardiologists responded to the survey and more than one-third of oncologists are using all of the seven proformas, especially in Europe and North America. The HFA/IC-OS baseline cardiovascular risk stratification, which has not been evaluated prospectively, is based on expert opinion and was published several months (August 2020) before the conduction of our survey in April–July 2021. The high rate of proformas utilization is likely to reflect the responders to the survey who are experts in cardio-oncology and early adopters of the HFA/IC-OS risk score within a year of publication (5). However, more than one-quarter of cardiologists and oncologists in the Middle East do not use baseline cardiovascular risk assessment. Efforts should be made in this region to increase awareness of the importance of baseline CV risk assessment at cancer diagnosis as recommended by the 2021 ESC guideline for the management of heart failure [class I], particularly in those patients with a history or risk factors for CVD, history of cardiotoxicity, or planned exposure to cardiotoxic cancer therapy (6). Moreover, baseline CV risk evaluation should be considered in all patients with cancer who are planning to receive cancer treatment with the potential to develop heart failure [class IIa] (6). More than half of the respondents, especially oncologists, consider hypertension as the main CVD to be treated among patients with cancer in three continents except in Europe, where heart failure was considered the main CVD. This regional variation may be explained by the fact that the oncologists in Europe are more likely to prescribe the treatment of hypertension among patients with cancer based on European Society of Medical Oncology (ESMO) consensus recommendations (7). Other surveys have also shown hypertension as the most common CVD according to the oncologists' opinion (71%), whereas heart failure is the most common one based on cardiologists' opinion (60.3%) (8). About two-thirds of the cardiologists and oncologists start antihypertensive agents for patients with cancer when BP is >140/90 mmHg mainly in the Middle East, Latin America and the Caribbean, and Europe, while in North America, the cutoff BP value is >130/80 mmHg. Regional variations may be related to the different cutoff normal values for the BP and values for initiation of antihypertensive agents in the general population in the American and European guidelines. The recent definition by the 2021 IC-OS consensus considered the normal BP ≤130/80 mmHg and the BP threshold to start an antihypertensive agent in patients with cancer is ≥140/90 mmHg in the absence of established CVD or when ASCVD risk is < 10% (9). The 2018 ESC/ESH guidelines also recommend the initiation of anti-hypertensive treatment for patients with cancer for BP ≥140/90 mmHg or when diastolic BP increases ≥20 mmHg from the baseline value (10). ACEI is the main antihypertensive agent for the treatment of hypertension not induced by cancer therapies followed by ARB. Both of these drugs are recommended by the guideline as first-line antihypertensive agents. In addition, evidence showed that ACEI and ARB have anticancer effects by acting on angiotensin-type 2 receptors; a similar mechanism for their antihypertensive effect (10–12). The regional variation was found in the management of hypertension induced by cancer therapy in Europe; unlike in other regions, ACEI and CCB are mainly used drugs. The later regional variation may be interpreted by the recommendations of the ESMO consensus and the consensus of Spanish specialized societies and associations encouraging to use a combination of ACEI and dihydropyridine CCB in the management of hypertension, particularly when the patient is receiving VEGF and when there is difficulty in the control of BP (7, 13). Atorvastatin is the main statin used by the survey respondents, particularly by oncologists. ESMO focused on two statins, which have clinical trial evidence on cardioprotection in patients with cancer, namely, pravastatin and atorvastatin, and this may explain the tendency of oncologists to use atorvastatin (7). Atorvastatin also has some emerging anticancer effects, which may increase its popularity in oncology, but the highly lipophilic nature of atorvastatin leads to more drug–drug interactions with many cancer therapies (14, 15). For patients with cancer and borderline LVEF, more than one-fifth of the respondents do not prescribe any cardioprotective agent, particularly in the Middle East may be because most of the respondents in this region are oncologists. Clinical inertia including lack of awareness of the guidelines and resistance to change the previous clinical practice can be a contributed factor to the limited prescribing of cardioprotective agents by the specialists in the emerging field of cardio-oncology (16). Cardioprotective agents including ACEI and BB are recommended among patients with cancer who developed cardiotoxicity with a reduction of LVEF to ≥40% but <50% (7). Also, this regimen may be used in the case of normal LVEF in patients who have a cardiovascular risk factor and planning to receive cancer therapy with known cardiotoxicity (7). Decision-making by the majority of the survey respondents worldwide was not affected by the COVID-19 pandemic. At the beginning of the COVID-19 pandemic, there was a concern regarding the detrimental effect of ACEI/ARB, which might worsen COVID-19 outcomes through upregulation of ACE2 receptor; a hypothesis that was later rejected by the growing evidence from real-world practice showed the beneficial effect of these drugs with no increase in mortality (17–22). Therefore, the respondents are following the guideline-directed therapy, which is based on firm evidence rather than a hypothesis of the association between ACEI/ARB and COVID-19. Regarding the selection of an anticoagulant for the management of VTE in patients with cancer, one-third of the survey responders prescribe apixaban. The 2020 American Society of Clinical Oncology (ASCO) guideline recommends low-molecular-weight heparin (LMWH), edoxaban, or rivaroxaban for the management of VTE in patients with cancer, with caution in the case of NOAC drug–drug interaction and increased risk of bleeding with GI and GU malignancies (23). While the 2020 National Comprehensive Cancer Network guideline recommends apixaban, edoxaban, or rivaroxaban as first-line and preferred over LMWH (23). In case of infection with COVID-19, it was found that enoxaparin is the most commonly prescribed anticoagulant among patients with cancer followed by apixaban; however, still, the use of enoxaparin is higher among oncologists, and apixaban is higher to be prescribed by cardiologists. Of note, in Europe, the use of edoxaban decreased and the use of enoxaparin increased for patients with cancer and COVID-19. LMWH is advised for patients with cancer and COVID-19 infection to reduce the risk of thromboembolism based on renal function (24). In addition to the aforementioned, the selection of anticoagulants in patients with cancer is complex, which involves patient's preferences, variation in the prognosis of each type of cancer, comorbid disease, drug–drug interactions, body weight, and impact of individual anticoagulant on VTE recurrence, bleeding, and mortality (25, 26).

In summary, the survey shows for the first time the worldwide regional variations in the management of CVD in cardio-oncology including the use of baseline cardiovascular risk stratification and the limitation in the use of cardioprotective agents. We recommend the adoption of international cardio-oncology guidelines, the inclusion of cardio-oncology training courses in the syllabus of cardiology fellows-in-training, and multidisciplinary teamwork at the cardio-oncology clinics to improve the standard of care and management of CVD in patients with cancer.

The study is limited by the use of an online survey with a small sample size of respondents. The study is biased by the large number of survey respondents who are experts in cardio-oncology and members of IC-OS.

In conclusion, more than three-quarters of cardiologists and oncologists are using HFA/IC-OS proformas within the first year of the publication. Our survey showed regional variations in the management of CVD in different continents. The use of cardioprotective agents is limited mainly in the Middle East; a call for action is required. The COVID-19 pandemic affected daily practice in the selection and switching of cardiovascular drugs, including ACEI/ARB and the choice of anticoagulants. Regional variations showed that heart failure is the main CVD among patients with cancer, compared to other continents in which hypertension is the number one CVD. Other regional variations were noted regarding the cutoff value for treating hypertension. Apixaban is the first anticoagulant used among patients with cancer; however, after the COVID-19 pandemic, enoxaparin has been used as the first option.

## Data availability statement

The original contributions presented in the study are included in the article/Supplementary material, further inquiries can be directed to the corresponding author/s.

## Ethics statement

Ethical review and approval was not required for this study in accordance with the local legislation and institutional requirements.

## Author contributions

HF, IY, AL, and SD designed the study survey and had substantial role in writing the manuscript. IY performed the statistical analysis. All authors reviewed, edited, and approved the final manuscript for publication.

## Funding

AL is supported by the Leducq Foundation Transatlantic Network of Cardio-Oncology.

## Conflict of interest

Author DL was employed by IC-OS. Author SD reports honoraria for CME from Novartis, Astra Zeneca. The remaining authors declare that the research was conducted in the absence of any commercial or financial relationships that could be construed as a potential conflict of interest.

## Publisher's note

All claims expressed in this article are solely those of the authors and do not necessarily represent those of their affiliated organizations, or those of the publisher, the editors and the reviewers. Any product that may be evaluated in this article, or claim that may be made by its manufacturer, is not guaranteed or endorsed by the publisher.

## Abbreviations

ACEI: angiotensin converting enzyme inhibitor
ARB: angiotensin receptor blocker
ARNI: angiotensin receptor-neprilysin inhibitors
BB: beta blocker
CCB: calcium channel blocker
CV: cardiovascular
CVD: cardiovascular disease
HFA: Heart Failure Association
HFrEF: heart failure with reduced ejection fraction
IC-OS: International Cardio-Oncology Society
LVEF: left ventricular ejection fraction
RAAS: renin-angiotensin-aldosterone system
VTE: venous thromboembolic events.
