# Supplementary material for: Global pattern of cardiovascular disease management in patients with cancer and impact of COVID-19 on drug selection: IRAQ—IC-OS survey-based study
Source: Front Cardiovasc Med. 2022 Sep 21;9:979631. doi: 10.3389/fcvm.2022.979631 (PMC9532627; doi:10.3389/fcvm.2022.979631)
Supplement: Supplementary file 1 [file Data_Sheet_1.docx]

**Supplementary Figure 1: Blood pressure cutoff value to treat hypertension among patients with cancer in different continents**

Cutoff blood pressure value for the management of hypertension among patients with cancer is >140/90 globally, unlike in North America, where the main cutoff value is >130/80

**Supplementary Table 1: Iraq – IC-OS Online Survey**

| 1. What is your specialty:    1. Cardiologist    2. Medical (solid or heme) Oncologist    3. Radiation Oncologist    4. Cardiology resident/fellow    5. Oncology resident/fellow 2. What is the region of your current practice?    1. Africa    2. Asia    3. Europe    4. Latin America and the Caribbean    5. Middle East    6. North America    7. Oceania 3. Years of practice in cardiology/oncology/hematology    1. 1-5 years    2. 6-10 years    3. > 10 years 4. Are you prescribing cardiovascular drugs for cancer patients?    1. Yes    2. No 5. Do you monitor cardiovascular drugs that you prescribe for cancer patients during follow up (e.g. ECG, renal function test, serum electrolytes)?    1. Yes    2. No 6. Which is the most common cardiovascular disease you are treating among cancer patients?    1. Hypertension    2. Heart failure    3. Thrombosis    4. Ischemic heart disease    5. Arrhythmias    6. Other [please specify] 7. At which blood pressure do you initiate anti-hypertensive agents among cancer patients?    1. >120/80    2. >130/80    3. >140/90    4. Other [please specify] 8. Which is the most common drug you prescribe for hypertension (not induced by cancer therapy) for cancer patients?    1. ACEI [*Angiotensin converting enzyme inhibitor]*    2. ARB [*Angiotensin receptor blocker]*    3. CCB [*Calcium channel blocker*]    4. Thiazide diuretic    5. Combined tablet containing ACEI    6. Combined tablet containing ARB    7. Other [please specify] 9. Which is the most common drug you prescribe for hypertension (induced by tyrosine kinase inhibitor therapy) for cancer patients?    1. ACEI    2. ARB    3. CCB [*Calcium channel blocker*]    4. Thiazide diuretic    5. Combined tablet containing ACEI    6. Combined tablet containing ARB    7. Other [please specify] 10. Which is the most common anticoagulant you prescribe for VTE in cancer patients?     1. Enoxaparin     2. Unfractionated heparin     3. Warfarin     4. Apixaban     5. Dabigatran     6. Edoxaban     7. Rivaroxaban     8. Do not prescribe 11. Which is the most common drug you prescribe for dyslipidemia in cancer patients?     1. Atorvastatin     2. Rosuvastatin     3. Simvastatin     4. Pravastatin     5. Other [please specify] 12. Which is the most common renin-angiotensin-aldosterone blocker you prescribe for left ventricular dysfunction with reduced ejection fraction (HFrEF) in cancer patients?     1. ACEI     2. ARB     3. ARNI [*Angiotensin receptor-neprilysin inhibitor*]     4. Do not prescribe 13. In cancer patients who are at risk of cardiovascular disease [with borderline left ventricular ejection fraction (e.g. 50%), normal blood pressure and heart rate], which cardiovascular agent(s) do you prescribe?     1. ACEI     2. ARB     3. BB [*Beta blocker*]     4. ACEI + BB     5. ARB + BB     6. None     7. Other [please specify] 14. Do you check for drug-drug interaction when adding cardiovascular drugs to patients receiving cancer therapy?     1. Yes     2. No 15. Do you perform baseline cardiovascular risk stratification for cancer patients?     1. Yes, using proforma of Joint HFA/IC-OS position statement for anthracycline chemotherapy     2. Yes, using proforma of Joint HFA/IC-OS position statement for HER2-targeted cancer therapies     3. Yes, using proforma of Joint HFA/IC-OS position statement for vascular endothelial growth factor inhibitors     4. Yes, using proforma of Joint HFA/IC-OS position statement for multi-targeted kinase inhibitors for chronic myeloid leukaemia     5. Yes, using proforma of Joint HFA/IC-OS position statement for proteasome inhibitors and immunomodulatory agents for multiple myeloma     6. Yes, using proforma of Joint HFA/IC-OS position statement for combination RAF and MEK inhibitors     7. Yes, using pro forma of Joint HFA/IC-OS position statement for androgen deprivation therapies     8. Yes, all of the above     9. No, I don't perform baseline cardiovascular risk stratification for cancer patients     10. Other 16. Does COVID-19 pandemic impact on your decision while prescribing ACEI versus ARB to treat CVD among cancer patients?     1. Yes, now I am using ACEI instead of ARB more frequently     2. Yes, now I am using ARB instead of ACEI more frequently     3. No, COVID-19 doesn't affect on my practice     4. Other [please specify] 17. For cancer patients who are already on ACEI or ARB then infected with COVID-19, do you switch between ACEI and ARB?     1. Yes, switch ACEI to ARB     2. Yes, switch ARB to ACEI     3. No, keep the patient on his medication whether ACEI or ARB     4. Other 18. For cancer patients who are infected with COVID-19 pandemic and eligible for anticoagulant, which is the most anticoagulant you prescribe?     1. Enoxaparin     2. Unfractionated heparin     3. Warfarin     4. Apixaban     5. Dabigatran     6. Edoxaban     7. Rivaroxaban     8. Other [please specify] 19. Your email (your email will be kept confidential) 20. Your name (optional) 21. Do you have any other comment? |
| --- |

**Supplementary Table 2: Management of CVD in cardio-oncology patients**

|  | Overall  140 (100) | Cardiologist 102 (72.9) | Oncologist 38 (27.1) | |
| --- | --- | --- | --- | --- |
| The most common CVD | | | |  |
| Hypertension | 73 (52.10) | 47 (46.1) | 26 (68.4) | |
| Heart failure | 44 (31.40) | 42 (41.2) | 2 (5.3) | |
| Thrombosis | 9 (6.40) | 2 (2) | 7 (18.4) | |
| Ischemic heart disease | 8 (5.70) | 6 (5.9) | 2 (5.3) | |
| Arrhythmias | 4 (2.90) | 3 (2.9) | 1 (2.6) | |
| Other: Dyslipidemia | 2 (1.40) | 2 (2) | 0 (0) | |
| Blood pressure cutoff value to treat hypertension | | | | |
| >120/80 | 3 (2.10) | 2 (2) | 1 (2.6) | |
| >130/80 | 31 (22.10) | 28 (27.5) | 3 (7.9) | |
| >140/90 | 102 (72.90) | 69 (67.6) | 33 (86.8) | |
| Other | 4 (2.90) | 3 (2.9) | 1 (2.6) | |
| The most common treatment for hypertension (not induced by cancer therapy) | | | | |
| ACEI | 69 (49.30) | 54 (52.9) | 15 (39.5) | |
| ARB | 39 (27.90) | 29 (28.4) | 10 (26.3) | |
| CCB | 11 (7.90) | 6 (5.9) | 5 (13.2) | |
| Thiazide diuretic | 2 (1.40) | 2 (2) | 0 (0) | |
| Combination contains ACEI | 5 (3.60) | 4 (3.9) | 0 (0) | |
| Combination contains ARB | 10 (7.10) | 3 (2.9) | 7 (18.4) | |
| Other | 4 (2.90) | 4 (3.9) | 1 (2.6) | |
| The most common treatment for hypertension (induced by tyrosine kinase inhibitor therapy) | | | | |
| ACEI | 60 (42.90) | 45 (44.1) | 15 (39.5) | |
| ARB | 33 (23.60) | 23 (22.5) | 10 (26.3) | |
| CCB | 21 (15) | 15 (14.7) | 6 (15.8) | |
| Thiazide diuretic | 1 (0.70) | 0 (0) | 1 (2.6) | |
| Combination contains ACEI | 11 (7.90) | 11 (10.8) | 0 (0) | |
| Combination contains ARB | 11 (7.90) | 6 (5.9) | 5 (13.2) | |
| Other | 3 (2.10) | 2 (2) | 1 (2.6) | |
| The most common prescribed statin | | | | |
| Atorvastatin | 72 (51.40) | 46 (45.1) | 26 (68.4) | |
| Pravastatin | 0 (0) | 0 (0) | 0 (0) | |
| Rosuvastatin | 62 (44.30) | 53 (52) | 9 (23.7) | |
| Simvastatin | 4 (2.90) | 2 (2) | 2 (5.3) | |
| Pitavastatin | 1 (0.70) | 1 (1) | 0 (0) | |
| Other | 1 (0.70) | 0 (0) | 1 (2.6) | |
| The Most RASS inhibitor prescribed for HFrEF | | | | |
| ACEI | 79 (56.40) | 64 (62.7) | 15 (39.5) | |
| ARB | 23 (16.40) | 14 (13.7) | 9 (23.7) | |
| ARNI | 24 (17.10) | 24 (23.5) | 0 (0) | |
| Do not prescribe | 13 (9.30) | 0 (0) | 13 (34.2) | |
| Other: Consulting cardiologist | 1 (0.70) | 0 (0) | 1 (2.6) | |
| The most common cardioprotective agents prescribed for patients with cancer and borderline LVEF [LVEF 50%] | | | | |
| ACEI | 22 (15.70) | 17 (16.7) | 5 (13.2) | |
| ARB | 8 (5.70) | 3 (2.9) | 5 (13.2) | |
| BB | 21 (15) | 18 (17.6) | 3 (7.9) | |
| ACEI + BB | 43 (30.70) | 36 (35.3) | 7 (18.4) | |
| ARB + BB | 10 (7.10) | 9 (8.8) | 1 (2.6) | |
| None | 32 (22.90) | 16 (15.7) | 16 (42.1) | |
| Other | 4 (2.90) | 3 (2.9) | 1 (2.6) | |
| *ACEI=angiotensin converting enzyme inhibitor; ARB=angiotensin receptor blocker; ARNI=angiotensin receptor-neprilysin inhibitors; BB=beta blocker; CCB=calcium channel blocker; CVD=cardiovascular disease; LVEF=left ventricular ejection fraction* | | | | |
